# Characterisation of Hybrid Polymersome Vesicles Containing the Efflux Pumps NaAtm1 or P-Glycoprotein

**DOI:** 10.3390/polym12051049

**Published:** 2020-05-03

**Authors:** Sarah Rottet, Shagufta Iqbal, Paul A. Beales, Anran Lin, Jiwon Lee, Melanie Rug, Colin Scott, Richard Callaghan

**Affiliations:** 1 CSIRO Synthetic Biology Future Science Platform, GPO Box 1700, Acton, Canberra 2601, Australia; sarah.rottet@anu.edu.au (S.R.); colin.scott@csiro.au (C.S.); 2 Research School of Biology, and the Medical School, Australian National University, Canberra 2601, Australia; shagufta.iqbal@anu.edu.au (S.I.); u6502001@anu.edu.au (A.L.); 3 School of Chemistry Bragg Centre for Materials Research and Astbury Centre for Structural Molecular Biology, University of Leeds, Leeds LS2 9JT, UK; P.A.Beales@leeds.ac.uk; 4 Centre for Advanced Microscopy, Australian National University, Canberra 2601, Australia; joanne.lee@anu.edu.au (J.L.); melanie.rug@anu.edu.au (M.R.)

**Keywords:** ATP binding cassette, reconstitution, membrane protein, polymersomes, liposomes, poly (butadiene)-poly (ethylene oxide), block co-polymer

## Abstract

Investigative systems for purified membrane transporters are almost exclusively reliant on the use of phospholipid vesicles or liposomes. Liposomes provide an environment to support protein function; however, they also have numerous drawbacks and should not be considered as a “one-size fits all” system. The use of artificial vesicles comprising block co-polymers (polymersomes) offers considerable advantages in terms of structural stability; provision of sufficient lateral pressure; and low passive permeability, which is a particular issue for transport assays using hydrophobic compounds. The present investigation demonstrates strategies to reconstitute ATP binding cassette (ABC) transporters into hybrid vesicles combining phospholipids and the block co-polymer poly (butadiene)-poly (ethylene oxide). Two efflux pumps were chosen; namely the *Novosphingobium aromaticivorans* Atm1 protein and human P-glycoprotein (Pgp). Polymersomes were generated with one of two lipid partners, either purified palmitoyl-oleoyl-phosphatidylcholine, or a mixture of crude *E. coli* lipid extract and cholesterol. Hybrid polymersomes were characterised for size, structural homogeneity, stability to detergents, and permeability. Two transporters, NaAtm1 and P-gp, were successfully reconstituted into pre-formed and surfactant-destabilised hybrid polymersomes using a detergent adsorption strategy. Reconstitution of both proteins was confirmed by density gradient centrifugation and the hybrid polymersomes supported substrate dependent ATPase activity of both transporters. The hybrid polymersomes also displayed low passive permeability to a fluorescent probe (calcein acetomethoxyl-ester (C-AM)) and offer the potential for quantitative measurements of transport activity for hydrophobic compounds.

## 1. Introduction

Membrane transport proteins control the selective barrier between the cell interior and the surrounding environment. Their functions are to regulate the entry and exit of nutrients, metabolic waste, endobiotics, and xenobiotics. The regulated flux of compounds is essential to cellular integrity and vital to growth and survival. Often, investigating membrane transporters is done using whole cell or organelle-based systems, and these methods provide important information on overall function and regulation. The use of isolated (i.e., purified) transporters reveals molecular information on their often complex mechanisms, allows measurement of detailed kinetic parameters, and provides the prospect of generating high-resolution structural information.

Several decades of hard-won experience have demonstrated the difficulties of investigating isolated membrane transporters. Their amphiphilic structural organisation necessitates extraction from the membrane environment with detergents, and this single factor has dramatic consequences for protein stability, structural integrity, and functional competence [1,2,3]. Chromatographic separation of membrane proteins during purification can strip away essential lipids or accessory protein components that support overall transporter function. As a consequence, investigative systems for transporters must adequately reflect the composition and biophysical features found in the original, native membrane micro-environment to ensure retention of function.

The time-honoured system for functional studies involves the reconstitution of transporters into phospholipid vesicles (liposomes) [4,5]. Liposomes are spherical structures encased by a phospholipid bilayer and contain an aqueous lumen. Typically, standard liposomes have diameters in the range 100–400 nm, although giant unilamellar vesicles (diameter 1–5 µm) are increasingly used for specialised applications [6]. The structures provide a rudimentary option to mimic natural cellular membranes, and they can be generated using myriad lipid components. This system has served biochemists well for many decades; however, they are characterised by a number of drawbacks. Liposomes are restricted to comparatively low lipid/protein ratios, which results in an environment with reduced lateral pressures and high fluidity [7]. Liposomes display inherent high permeability to hydrophobic compounds [8,9], which renders measurements on drug transport difficult, an issue demonstrated for the transporter P-glycoprotein (P-gp) [6,10]. It appears that the reconstitution of an integral membrane protein imparts further leakiness of the liposome bilayer to the luminal components. Therefore, measuring transport parameters for proteins dependent on the presence of a stable trans-bilayer ionic gradient (e.g., pfCRT) is also difficult.

Vesicles comprising synthetic polymers have increasingly been viewed as a viable alternative to liposomes; in particular, block co-polymers are able to generate the amphiphilic layout of natural membranes [11,12,13]. The polymer vesicles (hybrid and homogeneous) have been applied in numerous areas of research including protein biochemistry, drug delivery, and nanotechnology, as indicated in these references. Co-polymers refer to a composition containing two or more monomers and blocks correspond to short segments of the polymer comprising repeats of a single monomer. Vesicles comprising block co-polymers are referred to as polymersomes and offer a number of distinctive features and advantages to liposomes [13,14,15]. Segments of the block co-polymers exhibit extensive contacts and interdigitation, which generates low fluidity and a high degree of lateral pressure. These features also result in the formation of leaflets with low permeability to both ionic and organic biological molecules. Polymersomes are inherently more stable and require considerably larger torsional and shear force to rupture [14,16]. Finally, the almost infinite array of possible components enables the researcher to tailor the physical features to specific applications.

The application of polymersomes has been confined to a relatively small selection of proteins [17,18], and they have not yet been applied to important families such as the ATP binding cassette (ABC) proteins. ABC transporters mediate the translocation of an enormous array of bio-molecules using distinct transport processes, and thereby display considerable individuality and nuanced requirements to mediate function. Consequently, the polymersome reconstitution system will require optimisation to render it a generic tool for ABC proteins. For example, vesicles comprising block co-polymers display considerably greater thickness (5–40 nm) compared with conventional phospholipid bilayers (3–5 nm) [12]. The polymer membranes thus have a mismatch with the dimensions of ABC proteins. Provision of lateral constraint is beneficial for protein function, however, an excessive rigidity in polymersomes may prevent the numerous conformational transitions inherent to transporter activity [19]. Membrane proteins often display direct interactions with the lipid milieu and require specific annular components, some of which may not be replicated by a polymer environment [20,21].

Hybrid lipid–polymersomes offer a potential compromise between these two systems. The polymer component provides low permeability and high stability, while the glycerolipids provide a more natural annular environment and compatible bilayer dimension. The current investigation optimises the reconstitution of two distinct ABC transporters into hybrid polymersomes. The first, P-glycoprotein (P-gp), is a large eukaryotic efflux pump that transports an astonishing array and number of hydrophobic drugs. Moreover, P-gp displays a notoriously specific dependence on lipid components in its annular region [10,22]. The second protein is the *Novosphingobium aromaticivorans* orthologue of Atm1 (NaAtm1). The NaAtm1 protein is a homodimeric unit that mediates the active efflux of toxic metals complexed to glutathione [23,24]. Hybrid polymersomes were characterised for physical features and the success of reconstitution was ultimately defined through retention of transporter activity.

## 2. Materials and Methods

### 2.1. Expression and Purification of P-gp

The plasmid pKSmdr containing cDNA (genbank accession: M14758) for MDR1 (P-glycoprotein) was a gift from Dr D. Gill, University of Oxford. The coding sequence for a C-terminal dodecahistidine tag was introduced by subconing the plasmid into pAlter^TM^ as described by Promega. The tagged P-glycoprotein (P-gp) was sub-cloned into the baculovirus transfer vector pBacPAK9 (Clontech) to generate pBP9-MDR. Recombinant baculovirus was produced by cotransfecting the *Spodoptera frugiperda* (Sf9) insect cells with pBacPAK6 viral DNA and pBP9-MDR vectors. High-titre baculovirus was produced by amplification in SF9 cells, as described [25]. For the expression of P-gp, *Trichoplusia ni* (High Five) insect cells (3 × 10^6^) were infected with high-titre baculovirus at multiplicity of infection (MOI) of 2. Three days post infection, the cells were centrifuged at 3000 g for 10 min and stored at −80 °C. The crude membranes were prepared by nitrogen cavitation followed by ultra-centrifugation as described [26] and stored at −80 °C.

The membranes were homogenised in solubilisation buffer (20 mM MOPS, 200 mM NaCl, 20% v/v glycerol pH 6.8) supplemented with 2% (w/v) dodecyl-maltoside (DDM) (Anatrace, OH, USA). The membranes were solubilised at a protein concentration of 5 mg/mL with vigorous stirring (4 °C, 120 min) and the soluble fraction was separated by ultracentrifugation at 29,000 *g* (20 min, 4 °C). The soluble fraction was loaded onto a pre-equilibrated 5 mL HisTrap (GE Healthcare Aus, Parramatta NSW) column with immobilized metal ion affinity chromatography (IMAC) buffer A (20 mM MOPS pH 6.8, 200 mM NaCl, 10% glycerol, 0.01% DDM) at a flow rate of 0.5 mL/min. A stepwise gradient of imidazole (0.04–1 M) was applied at a flow rate of 3 mL/min and 5 mL fractions were collected.

To reduce the imidazole concentration, fractions containing P-gp were exchanged into IMAC buffer using a HiPrep column (GE Healthcare Aus, Parramatta NSW) at a flow rate of 10 mL/min. P-gp containing fractions were pooled and concentrated using Amicon^®^ centrifugal filters with a 100 kDa molecular weight cutoff (Merck, Nth Ryde, NSW), snap-frozen in liquid nitrogen, and stored at −80 °C. The concentration of purified P-glycoprotein was estimated via densitometry of quantitative SDS-PAGE using a BSA standard, as described [25].

### 2.2. Expression and Purification of NaAtm1

The plasmid pJL-H6, containing NaAtm1, was a gift from Douglas Rees (Addgene plasmid # 78308; http://n2t.net/addgene:78308; RRID: Addgene_78308). Expression of NaAtm1 was carried out as described elsewhere [24]. Briefly, the construct was expressed overnight in *Escherichia coli* strain BL21 (DE3) at 37 °C in autoinduction media ZYM-5052 [27]. For protein purification, the cell pellet from a 0.5 L-culture was resuspended in 40 mL of lysis buffer (20 mM Tris pH 7.5, 0.1 M NaCl, 0.1 mg/mL lysozyme, 1 mM PMSF, 0.01 mg/mL DNaseI, and 1% (w/v) DDM) and incubated with rotation for 1 h at 4 °C. Cells were homogenized five times through a microfluidizer (M-110P, Microfluidics Corp.). Insoluble proteins and debris were removed by ultracentrifugation at 100,000 g (40 min) prior to loading onto a 5 mL HisTrap HP column. The column was pre-equilibrated with IMAC buffer B (20 mM Tris pH 7.5, 0.1 M NaCl, 0.1% (w/v) DDM) and a gradient from 12% to 40% (v/v) of IMAC buffer C (20 mM Tris pH 7.5, 0.1 M NaCl, 0.1% (w/v) DDM, 0.5 M imidazole) was applied. Imidazole was removed from NaAtm1 samples by buffer exchange using a Superdex 200 column (GE Healthcare Aus, Parramatta NSW) pre-equilibrated with IMAC buffer B. Fractions containing NaAtm1 were pooled and concentrated to approximately 4 mg/mL using Amicon^®^ centrifugal filters with a 100 kDa molecular weight cutoff. Purified NaAtm1 was snap-frozen in liquid nitrogen and stored at −80 °C.

### 2.3. Formation of Liposomes, Polymersomes, and Hybrid Vesicles

1-Palmitoyl-2-oleoyl-glycero-3-phosphocholine (POPC) (Avanti Polar Lipids, AL, USA) was maintained as a 100 mg/mL stock in (2:1) chloroform/methanol at −80 °C. The complex lipid mixture comprising a 4:1 ratio of total *E. coli* crude lipid extract and cholesterol was also stored as a 100 mg/mL stock in (2:1) chloroform/methanol at −80 °C. Poly (butadiene-*b*-ethylene oxide) from Polymer Source, Dorval Canada (1.2-*b*-0.6) was kept in identical solvent and storage conditions at a concentration of 47.3 mg/mL.

The appropriate proportions of block co-polymer and/or lipid were mixed and dried under vacuum to generate a thin film on glass tubes. The fluorescent probe L-phosphatidyl-ethanolamine-N-(lissamine rhodamine B sulphonyl) (Avanti Polar Lipids, AL, USA) from a 1 mg/mL stock was added to the mixture at 0.1 mol% before drying to ensure dispersion. The films were hydrated in low ionic strength buffer I (20 mM MOPS Ph 7.4, 10 mM NaCl) with gentle magnetic stirring over a 48 h period at 25 °C. Following hydration, the vesicles were subjected to five cycles of rapid freezing (dry-ice-ethanol bath) and thawing (55 °C). The multi-lamellar suspensions were extruded sequentially through 400 and 200 nm polycarbonate filters using an Avanti Mini-Extruder. Passage initially through the 400 nm filter was used to prevent high pressure caused by pore blockage. Maintaining samples at elevated temperatures assists with the extrusion process, as previously indicated [14].

### 2.4. Cryo-Electron Microscopy of Polymer and Lipid Vesicles

The Cryo-TEM technique was applied to visualise vesicular membrane structures. Holey carbon coated grids (400 mesh, EMS) were glow-discharged to make a hydrophilic surface and 4 µL of sample was applied to the grid. After 10 s of incubation time, the excess sample was gently blotted and immediately plunged into liquid ethane using a plunge freezer (gravity-fed plungers from EMBL). Following this, vitrified samples were transferred to the TEM (Hitachi 7100) microscope under liquid nitrogen and visualised at 100 kv with a side entry cryo-TEM holder. Images were acquired with a CCD camera (Ultrascan, Gatan).

### 2.5. Reconstitution of Transporters Using a Detergent Adsorption Strategy

In order to reconstitute the transporters, preformed vesicles (1 mL of 6.57 mM) were destabilized with DDM and incubated with gentle rotation at 20 °C. After ten minutes, 10–40 µg of protein was added to the destabilised vesicles and the mixture was incubated at 4 °C for 40 min with gentle rotation. Following incubation, protein reconstitution was initiated by detergent removal using sequential addition of pre-washed SM2-adsorbent Bio-beads (BioRad, Gladesville NSW). For the first cycle, 40 mg of Bio-beads was added to the protein–vesicle mixture and incubated with gentle rotation at 4 °C for 30 min. The mixture was transferred to a new tube containing 40 mg of Bio-beads and incubated for an hour. This was followed by two further rounds of 40 mg Bio-bead addition and six hours of incubation.

### 2.6. Dynamic Light Scattering of Vesicles

The hydrodynamic diameters of vesicles were determined by dynamic light scattering (DLS) using a Zetasizer Nano ZS (Malvern). Extruded vesicles were diluted eightfold in low ionic strength buffer I to a concentration of 0.8–1.0 µmol lipid/mL and transferred to 40 µL cuvettes (ZEN0040, Malvern). Samples obtained from the sucrose density gradients were measured without dilution. The scattered light was measured at a fixed backscatter angle of 173°. Four measurements for each sample were performed at 25 °C and the vesicle intensity-weighted diameter was reported as the average of the four measurements using cumulants analysis (Z-average). Without knowing the optical properties of hybrid vesicles, only the primary results (i.e., intensity-based) of a distribution analysis (average of four measurements) were reported [28].

### 2.7. Sucrose Density Centrifugation of Vesicles

Reconstituted vesicles were analysed by flotation on discontinuous sucrose density gradients, as described previously [29,30]. Typically, 200 µL of vesicles was brought to 40%–45% (w/v) sucrose in an appropriate volume of 60% (w/v) sucrose in low ionic strength buffer I. The gradients were formed by layering 500 µL of 30%, 20%, 10%, 5%, and 0% (w/v) sucrose above the vesicle fraction. The gradients were centrifuged overnight at 100,000 g in a SW60Ti rotor (Beckman) at 4 °C followed by fraction (250 µL) collection. Vesicle distribution was quantified by location of the fluorescent probe L-α-phosphatidylethanolamine-N-(lissamine rhodamine B sulphonyl) (Rh-PE). A sample of each fraction was transferred to a 96-well plate and fluorescence was measured using a microplate reader (Tecan Infinite Pro) with an excitation at 560 ± 5 nm and emission at 583 ± 5 nm. The remainder of each fraction was used to measure vesicle size distribution by DLS and the presence of protein by SDS-PAGE.

### 2.8. Destabilisation and Solubilisation of Vesicles with Detergent

Lipid and polymeric vesicles are sufficiently large to scatter light and their solubilisation to form optically clear mixed micelles was monitored spectroscopically [29,30]. Vesicles (liposome, polymersome, or hybrid) were suspended in low ionic strength buffer I in a 1 mL quartz silica cuvette. The optical density was measured at 500 nm (OD_500_) using a Shimadzu UV2600 dual beam spectrophotometer. Sequential low volumes of DDM (0.05–1 M stock) or TX-100 (1%–10% (v/v)) were added to the cuvettes and mixed thoroughly, and the OD_500_ was recorded. Continuous measurement of OD_500_ was undertaken to ensure that the signal stabilised prior to the subsequent addition of detergent. Detergent additions were done until the OD_500_ signal was clarified, indicating the formation of mixed micelles. Solubilisation was undertaken for vesicles containing varying proportions (0%–100%) of poly (butadiene)-poly (ethylene oxide) (PBd-PEO) at a concentration of 6.57 mM. Alternatively, vesicles were fixed to a composition of 50:50 lipid/polymer and the total concentration was altered (1.25–13.12 mM). The OD_500_ was plotted as a function of detergent concentration to estimate the point of vesicle saturation (C_sat_) with surfactant and the point of solubilisation (C_sol_).

### 2.9. Permeability of Vesicles to Calcein Acetomethoxyl-Ester (C-AM)

Lipid and or polymer films (6.57 µmoles) were generated as described above and hydrated in low ionic strength buffer I (1.0 mL) containing 5U of porcine carboxyl-esterase (Sigma-Aldrich, Castle Hill). Following the formation of esterase-encapsulated vesicles (see *2.3 Formation of Liposomes, Polymersomes, and Hybrid Vesicles*), the extruded vesicles were subjected to size-exclusion chromatography to remove non-encapsulated esterase. Briefly, 500 µL vesicle preparation was injected onto a Superdex 200 Increase 10/300 column at a flow rate of 0.5 mL/min in low ionic strength buffer I. Absorbance was monitored at 280 nm and samples containing vesicles (void fractions 9–10 mL) were transferred to ice. Non-encapsulated esterase eluted from the column at a volume of 12.2 mL.

Accumulation of C-AM in vesicles was measured using a Cary Eclipse Fluorometer (Agilent Technologies). Vesicles (330 µL) were added to quartz silica cuvettes and the fluorescence was measured at 37 °C. Spectral parameters were as follows: excitation wavelength λ_ex_ = 488 ± 5 nm and emission wavelength λ_em_ = 515 ± 5 nm. The non-fluorescent probe C-AM (12 µM) was added to cuvettes at time *t = 0*. C-AM entering the vesicle lumen will be rapidly converted to the highly fluorescent and charged molecule calcein. The appearance of calcein fluorescence was monitored continuously for a period of 30 min.

### 2.10. Colorimetric Detection of ATPase Activity

The ATPase activity of reconstituted transporters was measured by the liberation of inorganic P_i_ using a colorimetric assay [25,31]. Activity of reconstituted protein (0.1–0.5 µg) was measured in the presence of varying substrate concentrations; nicardipine for P-glycoprotein, or glutathione (GSH) and Ag-GSH for NaAtm1. The reconstituted protein was incubated with Na_2_ATP (2 mM) in ATPase assay buffer (50 mM Tris.HCl pH 7.4, 150 mM NH_4_Cl, 5 mM MgSO_4_, 0.02% (w/v) NaN_3_). After 40 min, the reaction was terminated with 12% (w/v) SDS and the colour development was measured at λ_750nm_. The potency (EC_50_) of the substrate and maximal ATPase activity (V_max_) was estimated by non-linear regression of Equation (2).

### 2.11. Quantitative and Statistical Analyses

The increase in calcein fluorescence (F(t)) was plotted as a function of time (t) and fitted by the following logistic function:

F(t) = k∗F_0_/(*e*^(−r∗t)^∗k − e^(−r∗t)^∗F_0_ + F_0_) (1)

where k is the maximal fluorescence and r = the initial rate of increase.

The rate of ATP hydrolysis (*v*) was plotted as a function of drug concentration ([D]) and fitted using the general dose–response relationship:

(2) $$v=v_{initial}+\left(v_{final}-v_{initial}\right)/\left(1+10^{\left(log10\left(EC_{50}-\left[D\right]\right)\right)}\right)$$

where *v*_initial_ is the activity in the absence of drug, *v*_final_ is the maximal activity observed, and the potency is defined by EC_50_.

Comparison between average values was done using Student’s t-test with a *p*-value less than 0.05 considered statistically significant. All curve-fitting was done using non-linear least squares regression using GraphPad Prism 5.

## 3. Results

### 3.1. Biophysical Characteristics of Polymersomes and Liposomes

The block co-polymer PBd-PEO (1.2-*b*-0.6) was used in the present investigation as it has been shown to support activity of several integral membrane proteins, including cytochrome *bo3* [18,32]. PBd-PEO was used in combination with two different lipid species, palmitoyl-oleoyl-phosphatidylcholine (POPC), or a 4:1 mixture of crude *E. coli* lipid extract and cholesterol (EcCL). POPC has been widely used as the lipid of choice in hybrid polymersomes and is a major constituent of natural membranes. However, the activity of many membrane transporters has highly specific lipid requirements. For example, the highest ATPase activity of purified P-gp is provided by the more complex EcCL mixture [10].

**Figure 1:**
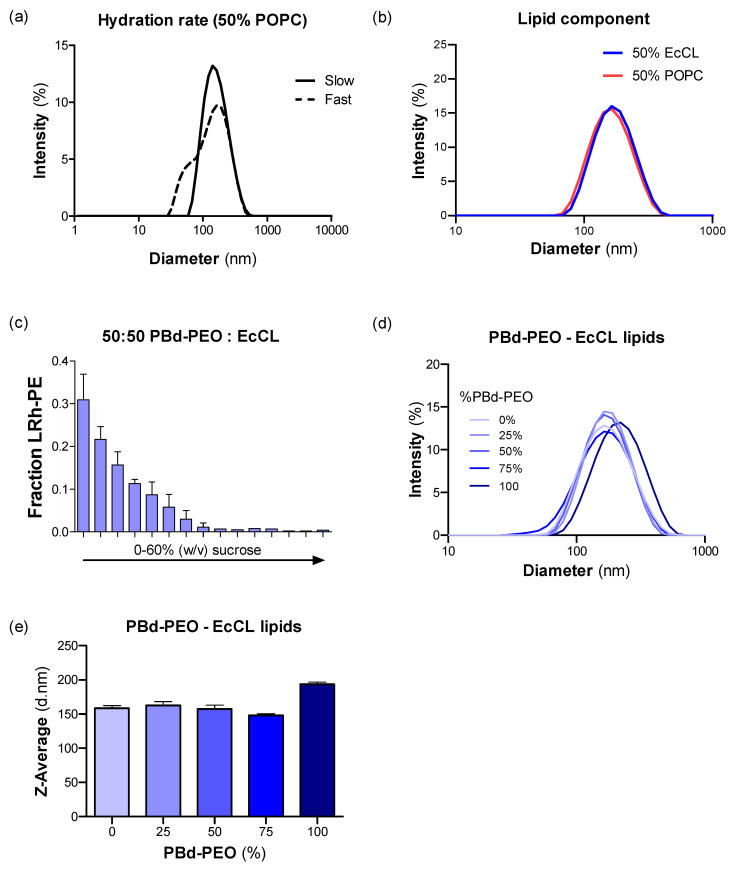
Physical features of hybrid polymersomes analysed using dynamic light scattering (DLS). (**a**) Hybrid vesicles comprising 50 mol% palmitoyl-oleoyl-phosphatidylcholine (POPC) and 50 mol% poly (butadiene)-poly (ethylene oxide) (PBd-PEO) were hydrated in buffer using two methods. The *fast* hydration involved vigorous stirring and heating to resuspend the lipid film. *Slow* hydration involved gentle stirring of the film in buffer over 48 h. DLS intensity profiles are shown for slow (*solid*) and fast (*dashed*) protocols. (**b**) DLS intensity profiles are shown for hybrid vesicles containing 50 mol% PBd-PEO. The remaining proportion comprised either POPC (*red*) or the crude *E. coli* lipid extract and cholesterol (EcCL) lipid mixture (*blue*). (**c**) The buoyancy of hybrid vesicles containing 50 mol% PBd-PEO and 50 mol% of EcCL lipid was measured in a sucrose density gradient. Vesicles contained 0.1 mol% of probe lipid L-α-phosphatidylethanolamine-N-(lissamine rhodamine B sulphonyl) (Rh-PE) to monitor their distribution through the gradient by fluorescence detection (λ_ex_ = 560 nm and λ_em_ = 583 nm). (**d**) DLS intensity profiles are shown for hybrid vesicles containing varying proportions of PBd-PEO and the crude EcCL lipid mixture. (**e**) The Z-average of the intensity profiles was used to estimate the hydrodynamic size of the vesicles shown in panel (**c**).

DLS provides insight into the size distribution of vesicles and was initially used to assess the two different hydration protocols. The rapid protocol, which involves multiple heating and mixing cycles over a 15 min period, resulted in a polydisperse population of vesicles, as shown in Figure 1a. In contrast, the slow protocol, in which samples were gently mixed over two days at room temperature, renders a monomodal population of vesicles.

The two lipid partners were combined with 50 mol% PBd-PEO and their DLS profiles were compared (Figure 1b). Despite its complex composition, the EcCL mixture did not alter the size or homogeneity of the vesicles when compared with POPC/PBd-PEO vesicles. Subsequent analyses on the efficiency of transporter insertion into vesicles rely on differential flotation on a sucrose density gradient. The distribution profile for hybrid EcCL/PBd-PEO vesicles (no transporter) is shown in Figure 1c for comparison with later distribution of proteo-vesicles.

The influence of varying proportions of EcCL (compared with PBd-PEO) on the vesicle size distribution after extrusion through 200 nm polycarbonate filters was compared using DLS profiles (Figure 1d,e). Neither the homogeneity, nor the diameter of DLS profiles was unaffected, apart from a small increase with vesicles comprising 100% PBd-PEO.

**Figure 2:**
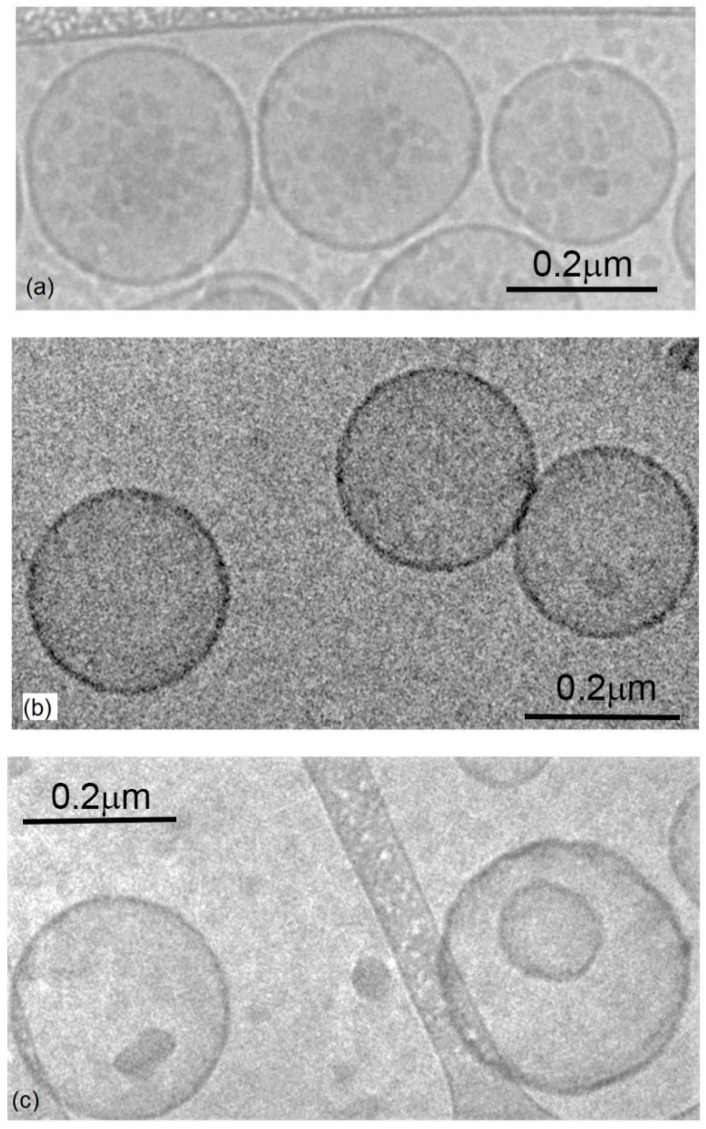
Structures of liposomes, hybrid vesicles, and polymersomes as determined using cryo-EM. Cryo-TEM images comparing (**a**) polymersome (PBd-PEO), (**b**) hybrid, and (**c**) liposomes (EcCL) in buffer indicates similar vesicle sizes. The vesicles appeared as unilamellar and spherical, with diameters typically in the range of 170–220 nm.

Overall, we showed that the hydration rate is important for homogeneity and that lipid partners have little impact on the biophysical properties of the vesicles. This was confirmed using transmission cryo-EM on liposomes (EcCL), polymersomes (PBd-PEO), and hybrid vesicles containing equal proportions of these two components (Figure 2). The micrographs demonstrated the homogeneity of vesicle sizes in accordance with the DLS analyses.

### 3.2. Solubilisation of Polymersomes and Liposomes—Influence of Detergent Class and Lipid Species

**Figure 3:**
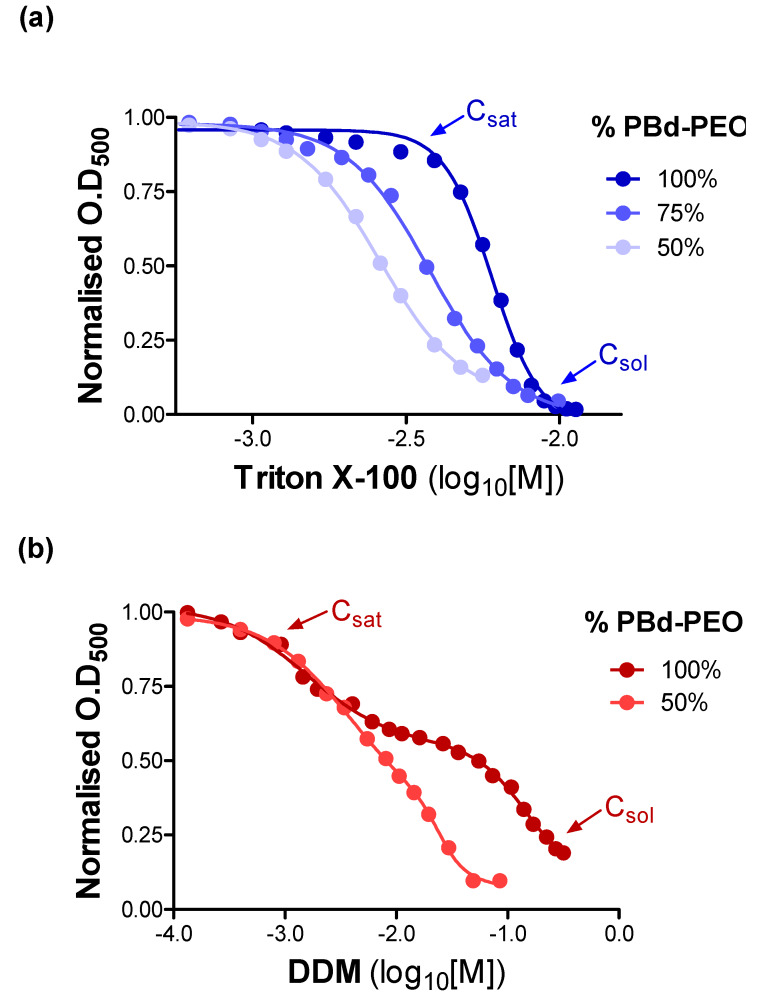
Solubilisation of vesicles containing varying proportions of PBd-PEO by the detergents Triton X-100 (TX-100) and dodecyl-maltoside (DDM). Absorbance (optical density (OD)) of vesicles was measured at 500 nm in a 1 cm path length quartz cuvette. Detergent was added and the signal was monitored continuously. All data were normalised to the optical density in the absence of detergent. The point of saturation with detergent (C_sat_) and the point of solubilisation (C_sol_) are shown for the highest polymer proportion in each panel for clarity. (**a**) Vesicles containing varying proportions of PBd-PEO (50%, 75%, and 100%) and EcCL were solubilised by the detergent Triton X-100. (**b**) Vesicles containing varying proportions of PBd-PEO (50% and 100%) and EcCL were solubilised by the detergent DDM.

The sensitivity to solubilisation of vesicles by detergent provides further characterisation of the biophysical properties of liposomes and polymersomes. Moreover, it is important to characterise the interactions between detergents and the lipid–polymer species to reveal the saturation points (C_sat_), and thereby ensure successful reconstitution. A detailed systematic account of the interaction between TX-100 and DDM, with multiple vesicle compositions, is provided in the Supplementary Files (Figures S1 and S2). The data in Figure 3 provide a snapshot of the distinct vesicle solubilisation profiles generated by TX-100 and DDM.

Figure 3a reveals a monophasic solubilisation profile for vesicles containing EcCL lipids in proportions between 0% and 50%. A higher proportion of PBd-PEO required considerably higher detergent concentrations to reach saturation (C_sat_) and solubilisation (C_sol_) points. This observation reflects an increase in stability that is conferred by the inclusion of block co-polymers in vesicles. In addition, the slope of the relationship describing TX-100 mediated solubilisation became steeper as the proportion of polymer increased, which demonstrates a narrow window between C_sat_ and C_sol_. This phase corresponds to bilayer destruction during the transition from lamellar to micellar structure and is consistent with TX-100 being a rapid solubiliser.

Figure 3b reveals a considerably more complex interaction between the alkyl-maltoside detergent DDM and vesicles containing PBd-PEO and EcCL. Only two of the compositions are displayed in this figure and the reader is directed to the Supplementary Data for a more comprehensive description. The solubilisation profiles were markedly broader compared with those with TX-100 and indicate a prolonged lamellar-micellar transition. Secondly, the solubilisation profiles were described by a two-phase equation, which is suggestive of multiple stages involved in the process of solubilisation by DDM. The complex phase behaviour was independent of the lipid species used in the vesicles; however, the relative proportions of lipid and polymer affected the process (Figure S2).

Clearly, the interaction between different classes of detergent and vesicles is not uniform and requires careful characterisation prior to embarking on protein reconstitution. Moreover, the interaction is highly dependent on the initial step of the process involving detergent intercalation into the vesicle outer leaflet [33,34,35]. TX-100 comprises an aromatic hydrophobic group and a hydrophilic polyethylene oxide chain that has chemical similarity to the block co-polymer and presumably aids intercalation. Conversely, DDM contains a bulky maltoside headgroup whose intercalation is likely to be energetically unfavourable in vesicle compositions with tight interactions between components.

### 3.3. Determining the Saturation and Solubilisation Points of Polymersomes and Liposomes

**Figure 4:**
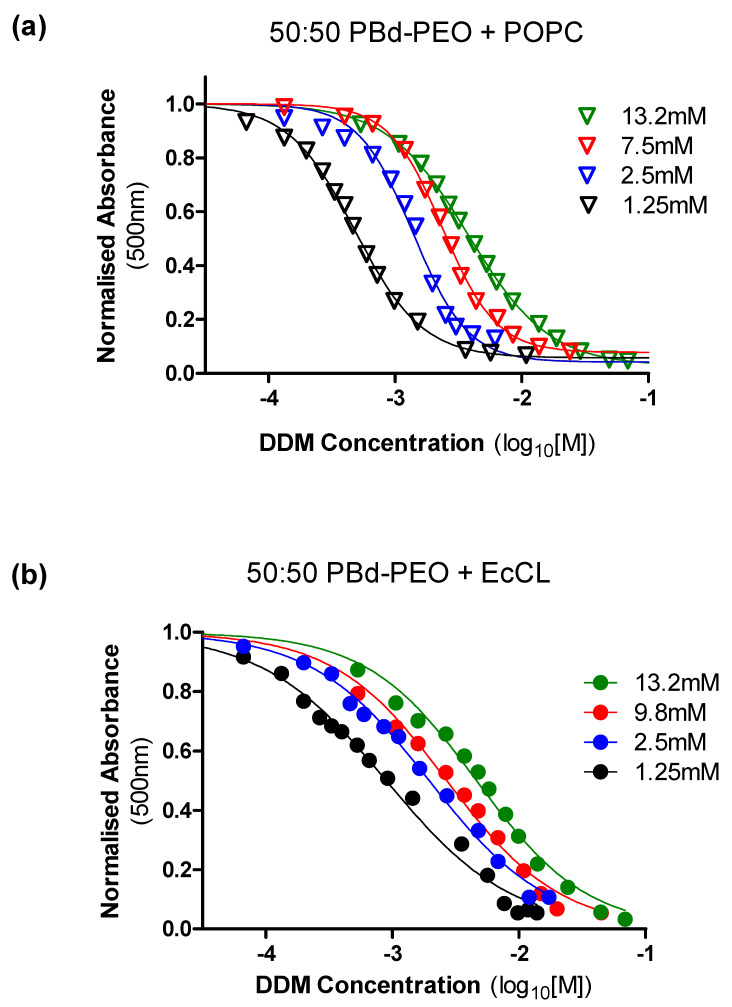
Effects of increased hybrid polymersome amount on solubilisation parameters. DDM (10^−4.5^ to 10^−1^ M) was used to solubilise varying concentrations (1.25–13.2 mM) of hybrid vesicles containing 50 mol% PBd-PEO and 50 mol% of lipid comprising either (**a**) POPC or (**b**) EcCL. Absorbance (optical density) was measured at 500 nm in a 1 cm path length quartz cuvette. Detergent was added and the signal was monitored continuously. Data were normalised to the optical density in the absence of detergent. The dose-response equation (variable slope) was fitted to the data using non-linear regression.

A number of membrane proteins, including P-gp [10], are more amenable to retention of function in the presence of DDM compared with TX-100. In addition, both P-gp and NaAtm1 were purified in DDM containing buffers. Therefore, despite its complex interaction with polymer containing vesicles, DDM was the first-choice detergent to destabilise vesicles in order to facilitate reconstitution. Figure 4 demonstrates the effects of varying the total amount of vesicle (50 mol% PBd-PEO in all cases) on the solubilising properties of DDM.

Vesicles containing POPC lipid (*panel a*) displayed a sharper gradient in the central portion of the curve and more clearly defined points of saturation and solubilisation by DDM. There was also a marked decrease in the sensitivity of POPC/PBd-PEO vesicles to DDM-mediated solubilisation as total vesicle concentration was increased. For example, DDM solubilisation of 1.25 mM hybrid vesicles was characterised by a C_sol_ = 3.6 mM and a vesicle concentration of 13.2 mM was associated with a C_sol_ = 48.9 mM. Hybrid vesicles containing the EcCL lipid mixture displayed markedly shallower solubilisation profiles by DDM (*panel b*), indicating a prolonged lamellar to micellar transition, regardless of the total vesicle concentration.

These profiles enable us to estimate the DDM concentration required to destabilise varying amounts of hybrid vesicles. Moreover, they reveal the relationship between the concentrations of detergent that vesicles are able to tolerate prior to breakdown of their structural integrity. This information is vital to determine the optimal conditions for transporter reconstitution into preformed vesicles of varying concentrations and compositions.

### 3.4. Permeability of Vesicles with Varied Lipid and Polymer Composition to C-AM

The effect of vesicle composition on permeability was investigated by a novel assay using the probe calcein acetomethoxyl-ester (C-AM). C-AM is a hydrophobic molecule that can readily cross cellular membranes. Once in the cytoplasm, the non-fluorescent C-AM is rapidly converted to the highly charged and fluorescent calcein molecule by non-specific carboxyl-esterases [36]. Our assay involves recreating this system by generating vesicles that contain porcine esterase within the lumen. Accordingly, diffusion of C-AM into the vesicle lumen will result in generation of calcein, with the rate of fluorescence increase providing a measure of membrane permeability.

**Figure 5:**
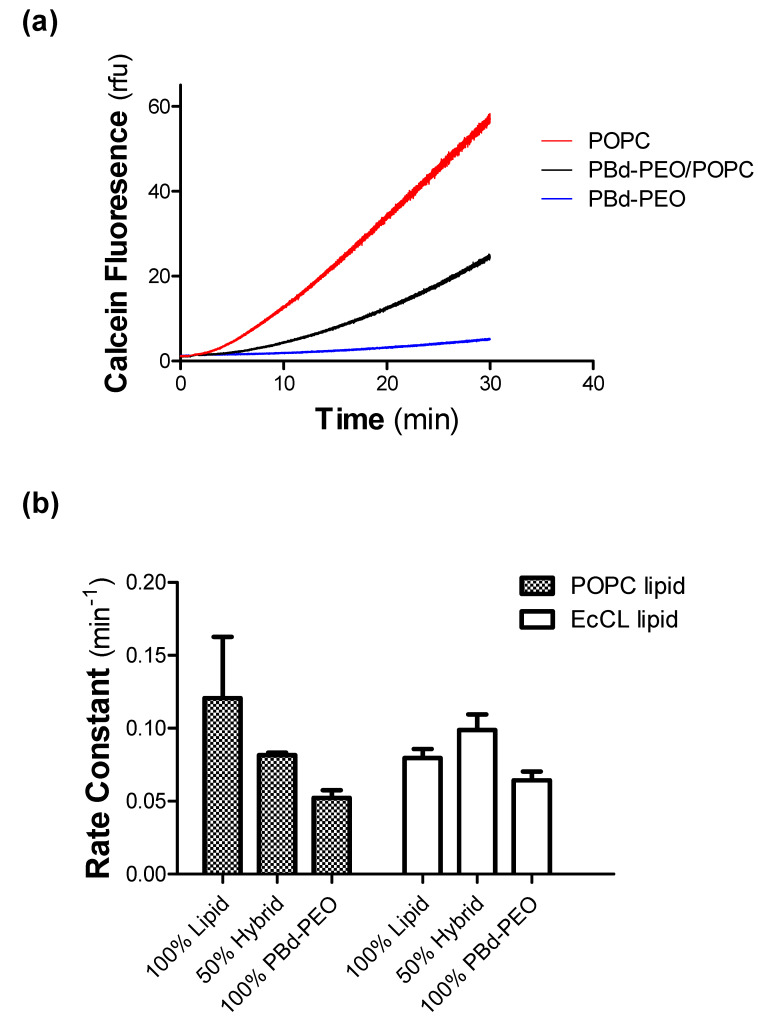
Permeability of two classes of polymersome to the fluorescent probe calcein acetomethoxyl-ester (C-AM). (**a**) The permeability of vesicles was assessed by the enzymatic (porcine carboxyl-esterase) production of fluorescent probe calcein from the non-fluorescent precursor C-AM within the lumen. Vesicles comprised 100 mol% POPC (*red trace*), 100 mol% PBd-PEO (*blue trace*), or hybrid vesicles with 50 mol% of both POPC and PBd-PEO (*black trace*). The C-AM probe (12 μM) was added to vesicles in quartz silica cuvettes at 37 °C. Fluorescence was measured continuously at λ_ex_ = 488 ± 5 nm and λ_em_ = 515 ± 5 nm. (**b**) The rate constant for the appearance of calcein fluoresence was determined by non-linear regression of logistic Equation (1). Rate constants were measured for vesicles whose lipid component was POPC (*hashed bars*) or EcCL (*clear bars*). Values represent the mean ± SEM obtained from three independent observations.

Figure 5a provides an example of calcein fluorescence as a function of time in vesicles composed of POPC (*red trace*), PBd-PEO (*blue trace*), and hybrid vesicles of POPC/PBd-PEO (*black trace*). All datasets were normalised to the fluorescence of Rh-PE, which was added to all vesicles at a proportion of 0.1 mol%. The esterase encapsulated POPC vesicles displayed the most rapid increase in the fluorescence, with the slowest rate of increase in PBd-PEO vesicles. A rate constant was obtained from the fluorescence profiles and the values are shown for all vesicle compositions in Figure 5b. Inclusion of PBd-PEO in the vesicles was associated with a reduced C-AM permeability compared with POPC liposomes. In contrast, vesicles composed of 100% EcCL or 100% PBd-PEO were characterised by similar rate constants for C-AM permeability. This suggests that the heterogeneity of phospholipids and the inclusion of cholesterol in the EcCL mixture enables tight packing within the bilayer and reducing C-AM diffusion. The hybrid EcCL/PBd-PEO (50:50) vesicles displayed an increased permeability to C-AM compared with 100% PBd-PEO, which may indicate that the lipid–polymer mixture was beset by inefficient packing properties of components. Clearly, distinct vesicle compositions of polymer and lipid will require individual characterisation of permeability properties.

### 3.5. Reconstitution of Functional NaAtm1 into Hybrid Polymersomes

**Figure 6:**
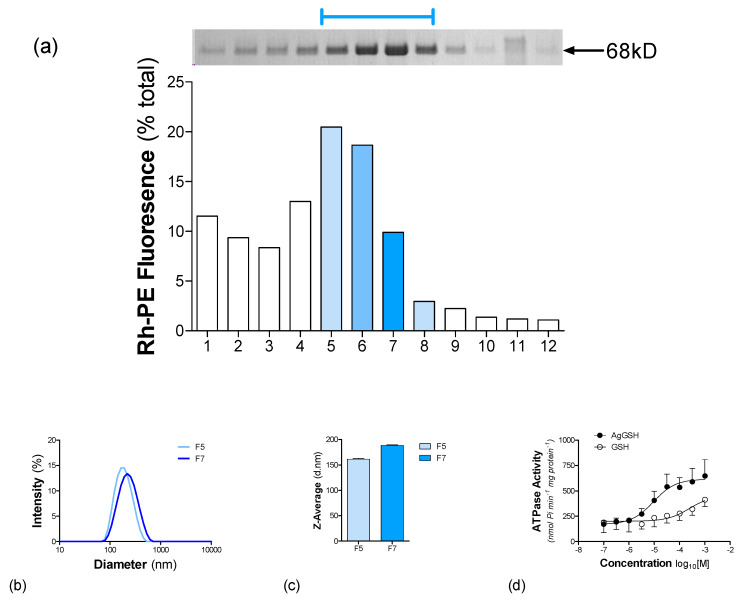
Effectiveness of reconstituting NaAtm1 into hybrid polymersomes. NaAtm1 was reconstituted into hybrid vesicles containing 50 mol% of each of PBd-PEO and POPC using detergent (DDM) adsorption. The success of reconstitution was assessed using sucrose density (0%–40% (w/v)) centrifugation (100,000 g, 18 h, 4 °C). Vesicles contained a small proportion (0.1 mol %) of the lipid probe Rh-PE. (**a**) The presence of Rh-PE fluorescence (λ_ex_ = 560 ± 5 nm and λ_em_ = 583 ± 5 nm) was monitored in each fraction collected from the gradient. The presence of NaAtm1 was detected in each fraction of the gradient using SDS-PAGE with AcquaStain Protein Gel Stain. The protein fractions (*upper panel*) were placed above their respective position within the sucrose gradient. (**b**) DLS intensity profiles measured for fractions F5 and F7 obtained from the sucrose density gradient. (**c**) The Z-average of the intensity profiles, used to estimate the hydrodynamic size of the vesicles. (**d**) The substrate stimulated ATPase activity was measured following reconstitution of NaAtm1 into hybrid vesicles of PBd-PEO (50 mol%) and POPC (50 mol%). ATPase activity was measured in the presence of a fixed amount of ATP (2 mM) and varying concentrations (10^−7^–10^−3^ M) of Ag-GSH (●) or GSH (⚬). Values represent the mean ± SEM obtained from three independent preparations and data were fitted by non-linear regression of the general dose-response Equation (2).

The POPC/PBd-PEO (50:50) hybrid vesicles were investigated for their ability to support the functional reconstitution of NaAtm1. The success of reconstitution was assessed by flotation in a sucrose density gradient and Figure 6a shows the distribution of NaAtm1 (*upper panel*) and Rh-PE (*lower panel*), which acts as a marker for the presence of vesicles. Following the complete removal of detergent, any non-reconstituted protein will form an insoluble aggregate found at the bottom of sucrose gradients [10]. NaAtm1 was located predominantly in fractions labelled F5–F8, and these contain 52% of the total amount of marker lipid. This co-localisation suggests successful reconstitution, with only a negligible fraction of NaAtm1 at the bottom of the sucrose gradient. The reconstituted vesicles have some heterogeneity in the amount of protein per unit of lipid–polymer; for example, the difference between F5 and F7. DLS analysis revealed symmetric profiles for both fractions (Figure 6b), although the average diameter in F7 (188 ± 1) was higher than that observed in F5 (161 ± 1) (Figure 6c). The reconstitution shown in Figure 6 used 40 µg of NaAtm1 with 3.29 µmol POPC/PBd-PEO. 

Does NaAtm1 reconstituted into hybrid polymersomes retain function? To answer this question, the ability of NaAtm1 to hydrolyse nucleotide was measured (Figure 6d) in the presence of increasing substrate concentrations. Ag-GSH and reduced glutathione have been shown to stimulate ATP hydrolysis by NaAtm1 [24] and were chosen as model substrates. In the absence of substrate, the basal ATPase activity of reconstituted NaAtm1 was 132 ± 33 nmol P_i_/min/mg protein (n = 4). In the presence of Ag-GSH, this activity was stimulated 4.3-fold to V_MAX_ = 701 ± 127 nmol P_i_/min/mg protein (n = 4); whereas GSH only stimulated the activity 2.2-fold to V_MAX_ = 413 ± 97 nmol P_i_/min/mg protein (n = 2). There are no comparable values currently available in the literature for NaAtm1; however, our preliminary work in POPC liposomes indicates an Ag-GSH stimulated activity with a V_MAX_ = 1107 ± 120 nmol P_i_/min/mg protein (n = 4).

In summary, NaAtm1 can be reconstituted into hybrid vesicles of POPC and PBd-PEO, where it retains substrate-stimulated (Ag-GSH and GSH) ATPase activity.

### 3.6. Reconstitution of Functional P-gp into Hybrid Polymersomes

**Figure 7:**
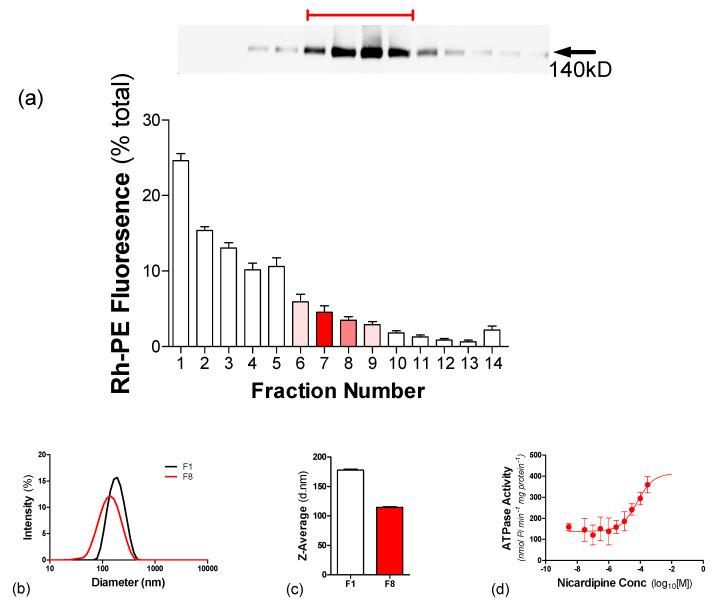
Effectiveness of reconstituting P-gp into hybrid polymersomes. P-gp was reconstituted into hybrid vesicles containing 50 mol% of each of PBd-PEO and EcCL using detergent (DDM) adsorption. The vesicles also contained a small proportion (0.1 mol%) of the lipid probe Rh-PE. (**a**) The presence of Rh-PE fluorescence (λ_ex_ = 560 ± 5 nm and λ_em_ = 583 ± 5 nm) was monitored in each fraction collected from the sucrose gradient. The presence of P-gp was detected in each fraction of the gradient using Western immune-blotting with an anti-His antibody. The protein fractions (*upper panel*) were placed above their respective position within the sucrose gradient. (**b**) DLS intensity profiles measured for fractions F1 (*top of gradient*) and F8 (*centre of gradient*) obtained from the sucrose density gradient. (**c**) The Z-average of the intensity profiles for F1 and F8, used to estimate the hydrodynamic size of the vesicles. (**d**) The substrate stimulated ATPase activity was measured following reconstitution of P-gp. ATPase activity was measured in the presence of a fixed amount of ATP (2 mM) and at varying concentrations (10^−9^–10^−4^ M) of Nicardipine. Values represent the mean ± SEM obtained from three independent preparations and data were fitted by non-linear regression of the general dose-response Equation (2).

P-gp was reconstituted into hybrid vesicles with POPC as the lipid component; however, this composition did not support ATPase activity. It is known that the ATPase activity of P-gp is highly specific and the EcCL mixture provides optimal retention of function [10]. Consequently, P-gp was reconstituted into hybrid vesicles of EcCL and PBd-PEO, with the protein and Rh-PE distributions shown in Figure 7a. The vesicles were destabilised with DDM and P-gp was localised to fractions F6–F9, with the majority in F8. There was a negligible amount of P-gp detected at the foot of the gradient and all detectable P-gp was localised with 17% of the total Rh-PE fluorescence. This indicates co-migration of P-gp with the vesicles, albeit with a high protein/vesicle ratio in these fractions. Figure 7b,c shows the DLS analysis of vesicles replete with P-gp (F8) and one devoid of protein (F1) for comparison. The former displayed a broader distribution profile and with an apparent vesicle diameter (114 ± 1 nm) that was lower than that observed for F1 (177 ± 2 nm). Figure 7d demonstrates that P-gp retained drug-stimulated ATPase activity in the hybrid PBd-PEO/EcCL vesicles. In the absence of drug, the rate of hydrolysis was 194 ± 69 nmol P_i_/min/mg, which corresponds to the basal activity. The ability of modulator drugs such as nicardipine to stimulate the ATPase activity of Pgp in purified, reconstituted liposomes has been well characterised [37]. In the presence of nicardipine, the ATPase activity was stimulated to 500 ± 81 nmol P_i_/min/mg, with a potency of 48 ± 12 μM (n = 3), demonstrating drug-stimulated nucleotide hydrolysis in hybrid polymersomes.

**Figure 8:**
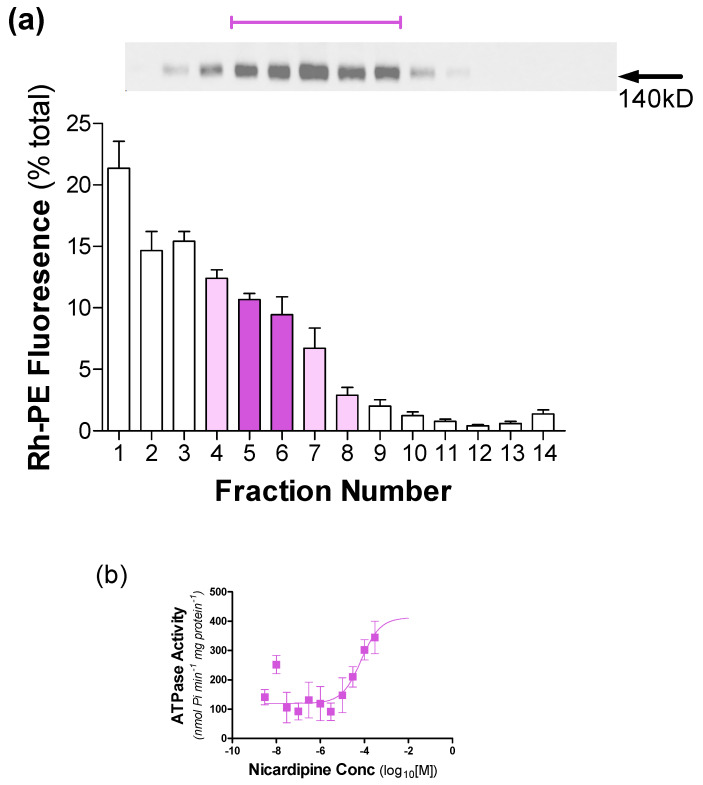
Effectiveness of reconstituting P-gp into hybrid polymersomes that were destabilised by TX-100. P-gp was reconstituted into hybrid vesicles containing 50 mol% of each of PBd-PEO and EcCL using detergent (TX-100) adsorption. The vesicles also contained a small proportion (0.1 mol %) of the lipid probe Rh-PE. (**a**) The presence of Rh-PE fluorescence (λ_ex_ = 560 ± 5 nm and λ_em_ = 583 ± 5 nm) was monitored in each fraction collected from the sucrose gradient. The presence of P-gp was detected in each fraction of the gradient using Western immuno-blotting with an anti-His antibody. The protein fractions (*upper panel*) were placed above their respective position within the sucrose gradient. (**b**) The substrate stimulated ATPase activity was measured following reconstitution of P-gp. ATPase activity was measured in the presence of a fixed amount of ATP (2 mM) and at varying concentrations (10^−9^–10^−4^ M) of Nicardipine. Values represent the mean ± SEM obtained from three independent preparations and data were fitted by non-linear regression of the general dose-response Equation (2).

TX-100 has a distinct interaction with PBd-PEO containing vesicles compared with DDM. Consequently, it was decided to assess whether destabilisation by TX-100 might prevent the high protein/lipid ratio in vesicles containing P-gp observed using DDM. The distribution of Rh-PE and protein following reconstitution is shown in Figure 8a and demonstrates P-gp localisation to fractions F4–F8. These fractions were associated with 42% of the total Rh-PE fluorescence, and thereby display a lower P-gp/vesicle ratio than that observed with DDM destabilisation in Figure 7. The underlying differences in P-gp reconstitution profiles with DDM versus TX-100 are unclear, but the distinct profiles were highly reproducible. Functional analysis was undertaken for P-gp (Figure 8b) and the reconstituted protein retained drug-stimulated ATPase activity. In the absence of drug, the activity was 114 ± 42 nmol P_i_/min/mg and could be stimulated to 403 ± 52 nmol P_i_/min/mg in the presence of nicardipine (EC_50_ = 47 ± 9 μM; n = 3).

In summary, P-gp could also be effectively reconstituted into hybrid vesicles containing PBd-PEO; however, the protein displayed a key dependence on a crude lipid mixture to retain function. In addition, the detergent used in the destabilisation process had a considerable influence on the protein/vesicle ratio obtained, although this had a minimal impact on drug-stimulated ATP hydrolysis.

## 4. Discussion

The low passive permeability of hybrid polymersomes offers enormous potential to investigate the vectorial movement of compounds by membrane transporters. This manuscript outlines a number of considerations involved for the successful reconstitution of two ABC transporters into hybrid polymersomes. The process of reconstitution requires that the target membrane structure is destabilised to facilitate transporter insertion [38] and, in the case of polymersomes, this is best characterised with the detergent TX-100 [14]. Unfortunately, numerous ABC transporters, including P-gp [10], display marked sensitivity to the class of detergent used during purification and reconstitution, with delipidation resulting in rapid and irreversible denaturation. Consequently, our initial choice of detergent was DDM, which is known to support P-gp function, and we adopted a strategy that involves using sub-solubilising detergent concentrations. Another key consideration is the lipid component used in hybrid polymersomes. P-gp is particularly sensitive to its lipid environment, with retention of activity only observed using crude lipid mixtures [10,39,40]. In contrast, the prokaryotic transporter NaAtm1 was decidedly more tolerant of conditions and components used for reconstitution. The limitations and differences highlighted above necessitate a detailed understanding of the interaction between protein, surfactant, and vesicle matrix in order to design a successful reconstitution strategy. This is particularly salient for the burgeoning area of polymer-based reconstitution systems.

To date, the majority of investigations with hybrid polymersomes [41,42,43], whether using micron (GUV) or nano (LUV) scale vesicles, have tended to incorporate a single synthetic lipid species with the block co-polymer. However, the activity of P-gp is best supported by a crude *E. coli* extract containing phospholipids and supplemented with cholesterol [10,39,44]. Despite the complexity of this EcCL lipid mixture, its use in generating hybrid polymersomes resulted in no discernible differences in heterogeneity, size, or density compared with vesicles containing the simple POPC lipid. It may be argued that the use of complex lipid mixtures provides more “suppleness” to deal with regions of elevated surface tension, high curvature, or the insertion of proteins. This may be supported by the finding that hybrid polymersomes containing POPC or EcCL as the lipid component displayed differences in their sensitivity to detergent-induced solubilisation. For example, DDM mediated solubilisation of vesicles containing synthetic POPC lipid had a sharper turbidity profile compared with those with EcCL. The central portion of the solubilisation phase diagram (i.e., stage 2) corresponds to co-existence of bilayer and micelle structures [30,35]. Similar observations were reported for the interaction of the related detergent octyl-glucoside with liposomes comprising POPC or *E. coli* lipid extract [34]. The authors concluded that lipids from the *E. coli* extract have a greater affinity for adopting bilayer phases compared with micelles. This preference was attributed to the significant proportion of lipids in the crude mixture that adopt structures with negative curvature, a property that is not compatible with standard micelle architecture [45].

Reconstitution of integral membrane proteins typically involves insertion into either mixed micelles or preformed vesicles. The former comprises mixing of detergent–lipid and detergent–protein–lipid micelles, with spontaneous bilayer formation and protein insertion occurring during the detergent removal phase [38,46,47]. However, this route may result in an unpredictable size distribution of proteoliposomes and the presence of multilamellar structures. This may be avoided by using preformed vesicles that are destabilised with detergent at sub-solubilisation concentrations. The pre-formed vesicle strategy requires extensive characterisation of detergent–vesicle interactions for which there is only limited information using polymer systems [18,48].

Consequently, to establish the point at which our vesicle types were destabilised (C_sat_), we characterised their transition from bilayer to micelle structures in response to TX-100 and DDM. This was monitored through turbidity measurements that reveal the three stages of solubilisation: (i) partitioning of detergent, (ii) bilayer disintegration, and (iii) mixed micelle formation [33,34,35]. In the case of DDM, it was difficult to assign a clear C_sat_-value owing to the observed multi-phase turbidity changes and shallow solubilisation profiles. The shallow slope and multiphase nature of vesicle solubilisation by DDM are likely to be caused by its slow rate of flipping to the inner leaflet [49]. This transbilayer flipping defines the nature of solubilisation for each detergent as described below. In addition to the multiphase solubilisation profiles, each addition of DDM to vesicles required considerably longer time periods than TX-100 to reach a steady turbidity level. It has been suggested [49] that the separation of phospholipid headgroups by the bulky maltoside moiety of DDM has a high energy barrier and slow kinetics.

Following on from this point, kinetic properties have been used to classify detergents into two categories [35]. Those in the first category (e.g., TX-100) rapidly flip from the outer to inner leaflet, leading to regions of high curvature and perforation of vesicle integrity. Complete solubilisation occurs through the subsequent formation of thread-like micelle structures. Detergents in the second category (e.g., DDM) contain headgroups that prevent, or slow the rate of, flipping to the inner bilayer leaflet. This creates a mass imbalance and curvature of the outer leaflet, leading to segments being pinched-off to generate mixed micelles.

Solubilisation of polymersome by detergents has considerably less mechanistic insight than the process in liposomes. The heterogeneity in polymer composition and interdigitation within vesicles suggest that solubilisation by detergents is unlikely to proceed with a unifying mechanism. Pata et al. [48] suggest a mechanism of solubilisation whereby, following initial perturbation, TX-100 moves through the “bilayer” and affects micellar extraction on both sides. However, other detergents may not penetrate the membrane and are more likely to prise apart the segments of blocks in the membrane.

The mechanism for solubilisation of hybrid polymersomes is likely to depend on how the lipid and polymer components are intermixed within the vesicles. Observations by Dao et al. [41] suggest the existence of non-vesicular structures, a mixture of pure liposomes and polymersomes, uniform intermixing of polymer and lipid, and lipid or polymer intra-vesicle domains. The distribution will depend on the method of formation, the relative proportions of components, the polymer class, and size (M_W_). Nam et al. [43] demonstrated, using GUVs, that the mixture of PBd-PEO and POPC generated well-mixed vesicles; however, it was possible to generate lipid-rich and polymer-rich domains. In a subsequent study [50], the authors used TX-100 to phase separate components, and this generated vesicle rupture and release of components. This investigation revealed differences in the interaction of TX-100 with lipid- or polymer-rich domains. We observed that, as the proportion of PBd-PEO was increased from 25% to 100%, the turbidity profiles to TX-100 moved from multi- to single phase and with a steeper slope. It is conceivable that this indicates a multi-stage solubilisation mechanism such as sequential solubilisation of lipid- and polymer-rich domains. The turbidity profiles for DDM did not reveal a similar pattern, and also varied according to the type of lipid used in the vesicles. It is noteworthy that the headgroup of TX-100 is polyethylene-oxide, which is chemically identical to the hydrophilic block of the polymer used in this investigation. This will ensure quite distinct partitioning behaviour to DDM.

As mentioned earlier, we have persisted with DDM despite its complex interaction with polymer containing vesicles because it does not lead to inactivation of P-gp or NaAtm1. Lambert et al. [49] demonstrated that sub-solubilising concentrations of DDM did not alter the morphology of egg phosphatidyl choline/phosphatidic acid vesicles, but resulted in increased size (and turbidity)—an observation we also found for POPC liposomes. During the vesicle disintegration stage of solubilisation, they observed multiple species including sealed and open vesicles, membrane sheets, and filaments. As the proportion of filaments increased, the solution became viscous and assumed a “gel-like” phase. We observed an apparently greater viscosity for each of the lipid–polymer compositions in the presence of DDM, but not when using TX-100. The formation of multiple intermediate structures may also contribute to the complex turbidity profiles observed with DDM.

Our reconstitution strategy used preformed liposomes destabilised with DDM, as it has been suggested to produce more asymmetry in protein orientation and greater retention of activity owing to the lower amounts of detergent added [38]. However, the process resulted in distinct outcomes for NaAtm1 and P-gp. While both were successfully reconstituted, the P-gp containing vesicles displayed a significantly higher protein/(lipid–polymer) ratio than that observed for NaAtm1. Previous investigations have demonstrated that P-gp purified in DDM from insect cells is obtained as monomer, dimer, and higher order oligomers, and that the distribution is altered by ionic conditions and amphiphiles [51,52]. In the present manuscript, switching to reconstitution of P-gp using TX-100 resulted in vesicles with a markedly lower protein/(lipid–polymer) ratio. It is tempting to speculate that TX-100 might disrupt P-gp–P-gp interactions and shift the equilibrium towards monomeric species. An early investigation with purified P-gp [53] demonstrated that P-gp solubilised in TX-100 was predominantly a monomer, whereas the zwitterionic detergent CHAPS produced a substantial proportion of dimers, trimers, and tetramers.

The ability to hydrolyse ATP and demonstration of substrate stimulated activity were chosen to assess the functional competence of P-gp and NaAtm1 following reconstitution into hybrid polymersomes. There are only scarce reports available on the activity of NaAtm1 [24] or its eukaryotic homologues [23], particularly for isolated protein. The yeast mitochondrial transporter Atm1p displayed a high basal activity that was stimulated 3–5-fold by GSH and a small selection of thiol containing compounds [23]. The ATPase activity of purified NaAtm1 [24] was stimulated to varying extents by reduced, oxidised, metal-chelated, and conjugated GSH derivatives. The overall catalytic rate was similar to our measurements and the greatest stimulation was found for Ag-GSH.

In contrast to Atm1, there are numerous reports detailing the ATPase activity of purified P-gp. The activity of P-gp in EcCL proteoliposomes is in the range of 200–1000 nmol P_i_/min/mg, with the upper and lower values signifying basal and drug-stimulated activity [25,37,54]. The catalytic rate in hybrid polymersomes was within this broad range and also retained a critical dependence on the lipid species used. The stimulation of ATP hydrolysis by nicardipine of 3–4-fold was retained; however, the reported values for EC_50_ are 1–4 µM [25,37]. This is considerably lower than the values obtained in hybrid polymersomes (Figure 7 and Figure 8). That P-gp extracts substrates directly from the membrane leaflet is a well characterised property, using multiple investigative approaches [36,55]. It is thus possible that the reduced potency of nicardipine in hybrid polymersomes reflects a lower insertion into the vesicle membrane, which effectively reduces the biophase concentration. Alternatively, the kinetics of conformational transitions may have been retarded in the more densely packed environment of a polymer-membrane mimetic. Conformational restrictions have been observed for the styrene–maleic acid (SMA)-nanodisc system with several ABC transporters [56]. However, the SMA-nanodiscs have a distinct architecture and lateral pressure that is markedly higher than in vesicles. Moreover, the more rigid/ordered environment of hybrid polymersomes may not fully permit interdomain communication, which is essential for ATP hydrolysis by both P-gp and NaAtm1.

In summary, ABC transporters can be reconstituted into polymer containing vesicles using the more compatible detergent DDM. Moreover, the conditions used in this manuscript ensured retention of function for both P-gp and NaAtm1. The key factor in reconstitution is the interaction between the detergent of choice with the specific lipid–polymer composition. It is also worth noting that the insertion of large integral membrane proteins will generate pronounced effects on the stability, packing, and biophysical properties of vesicles [34]. We observed this for the two efflux pumps, P-gp and NaAtm1. Our advice before embarking on reconstitution of transporters into hybrid polymersomes is to generate an understanding of the complex interactions between all components of the system.

## Supplementary Materials

The following are available online at https://www.mdpi.com/2073-4360/12/5/1049/s1, Figure S1: Solubilisation of two classes of hybrid polymersome by Triton X-100, Figure S2: Solubilisation of two classes of hybrid polymersome by dodecyl-β-maltoside.

## References

[1] Rothnie A.J. (2016). Detergent-Free Membrane Protein Purification. Heterologous Expression of Membrane Proteins.

[2] Seddon A.M., Curnow P., Booth P.J. (2004). Membrane proteins, lipids and detergents: Not just a soap opera. Biochim. Biophys. Acta.

[3] Smirnova I.A., Ädelroth P., Brzezinski P. (2018). Extraction and liposome reconstitution of membrane proteins with their native lipids without the use of detergents. Sci. Rep..

[4] Johnson Z.L., Lee S.-Y. (2015). Liposome Reconstitution and Transport Assay for Recombinant Transporters. Methods Enzymol..

[5] Skrzypek R., Iqbal S., Callaghan R. (2018). Methods of reconstitution to investigate membrane protein function. Methods.

[6] Horger K.S., Liu H., Rao D.K., Shukla S., Sept D., Ambudkar S.V., Mayer M. (2014). Hydrogel-assisted functional reconstitution of human P-glycoprotein (ABCB1) in giant liposomes. Biochim. Biophys. Acta.

[7] Curnow P., Lorch M., Charalambous K., Booth P.J. (2004). The Reconstitution and Activity of the Small Multidrug Transporter EmrE is Modulated by Non-bilayer Lipid Composition. J. Mol. Biol..

[8] Santos H.D.L., Lopes M.L., Maggio B., Ciancaglini P. (2005). Na,K-ATPase reconstituted in liposomes: Effects of lipid composition on hydrolytic activity and enzyme orientation. Colloids Surf. B Biointerfaces.

[9] Labelle E.F., Racker E. (1977). Cholesterol stimulation of penetration of unilamellar liposomes by hydrophobic compounds. J. Membr. Biol..

[10] Callaghan R., Berridge G., Ferry D.R., Higgins C.F. (1997). The functional purification of P-glycoprotein is dependent on maintenance of a lipid–protein interface. Biochim. Biophys. Acta.

[11] Blanazs A., Armes S.P., Ryan A. (2009). Self-Assembled Block Copolymer Aggregates: From Micelles to Vesicles and their Biological Applications. Macromol. Rapid Commun..

[12] Palivan C.G., Goers R., Najer A., Zhang X., Car A., Meier W.P. (2016). Bioinspired polymer vesicles and membranes for biological and medical applications. Chem. Soc. Rev..

[13] Rideau E., Dimova R., Schwille P., Wurm F.R., Landfester K. (2018). Liposomes and polymersomes: A comparative review towards cell mimicking. Chem. Soc. Rev..

[14] Beales P.A., Khan S., Muench S.P., Jeuken L.J.C. (2017). Durable vesicles for reconstitution of membrane proteins in biotechnology. Biochem. Soc. Trans..

[15] Schulz M., Binder W.H. (2015). Mixed Hybrid Lipid/Polymer Vesicles as a Novel Membrane Platform. Macromol. Rapid Commun..

[16] Garni M., Thamboo S., Schoenenberger C.-A., Palivan C.G. (2017). Biopores/membrane proteins in synthetic polymer membranes. Biochim. Biophys. Acta.

[17] Goers R., Thoma J., Ritzmann N., Di Silvestro A., Alter C., Gunkel-Grabole G., Fotiadis D., Müller D.J., Meier W.P. (2018). Optimized reconstitution of membrane proteins into synthetic membranes. Commun. Chem..

[18] Seneviratne R., Khan S., Moscrop E., Rappolt M., Muench S.P., Jeuken L., Beales P. (2018). A reconstitution method for integral membrane proteins in hybrid lipid-polymer vesicles for enhanced functional durability. Methods.

[19] Rodríguez-García R., Mell M., López-Montero I., Netzel J., Hellweg T., Monroy F. (2011). Polymersomes: Smart vesicles of tunable rigidity and permeability. Soft Matter.

[20] Barrantes F.J., Bermúdez V., Borroni M.V., Antollini S.S., Pediconi M.F., Baier J.C., Bonini I., Gallegos C., Roccamo A.M., Valles A.S. (2009). Boundary Lipids in The Nicotinic Acetylcholine Receptor Microenvironment. J. Mol. Neurosci..

[21] Lee A.G. (2003). Lipid–protein interactions in biological membranes: A structural perspective. Biochim. Biophys. Acta.

[22] Domicevica L., Koldsø H., Biggin P.C. (2018). Multiscale molecular dynamics simulations of lipid interactions with P-glycoprotein in a complex membrane. J. Mol. Gr. Model..

[23] Kuhnke G., Neumann K., Mühlenhoff U., Lill R. (2006). Stimulation of the ATPase activity of the yeast mitochondrial ABC transporter Atm1p by thiol compounds. Mol. Membr. Biol..

[24] Lee J.Y., Yang J.G., Zhitnitsky D., Lewinson O., Rees D.C. (2014). Structural Basis for Heavy Metal Detoxification by an Atm1-Type ABC Exporter. Science.

[25] Crowley E., O’Mara M.L., Reynolds C., Tieleman D.P., Storm J., Kerr I.D., Callaghan R. (2009). Transmembrane Helix 12 Modulates Progression of the ATP Catalytic Cycle in ABCB1. Biochemistry.

[26] Taylor A.M., Storm J., Soceneantu L., Linton K.J., Gabriel M., Martin C., Woodhouse J., Blott E., Higgins C.F., Callaghan R. (2001). Detailed characterization of cysteine-less P-glycoprotein reveals subtle pharmacological differences in function from wild-type protein. Br. J. Pharmacol..

[27] Studier F.W. (2005). Protein production by auto-induction in high-density shaking cultures. Protein Expr. Purif..

[28] Bhattacharjee S. (2016). DLS and zeta potential—What they are and what they are not?. J. Control. Release.

[29] Angrand M., Briolay A., Ronzon F., Roux B. (1997). Detergent-Mediated Reconstitution of a Glycosyl-Phosphatidylinositol-Protein into Liposomes. JBIC J. Biol. Inorg. Chem..

[30] Rigaud J.L., Paternostre M.T., Bluzat A. (1988). Mechanisms of membrane protein insertion into liposomes during reconstitution procedures involving the use of detergents. 2. Incorporation of the light-driven proton pump bacteriorhodopsin. Biochemistry.

[31] Chifflet S., Torriglia A., Chiesa R., Tolosa S. (1988). A method for the determination of inorganic phosphate in the presence of labile organic phosphate and high concentrations of protein: Application to lens ATPases. Anal. Biochem..

[32] Khan S., Li M., Muench S.P., Jeuken L.J.C., Beales P.A. (2016). Durable proteo-hybrid vesicles for the extended functional lifetime of membrane proteins in bionanotechnology. Chem. Commun..

[33] Clark S.T., Arras M.M., Sarles S.A., Frymier P.D. (2020). Lipid shape determination of detergent solubilization in mixed-lipid liposomes. Colloids Surf. B Biointerfaces.

[34] Krylova O.O., Jahnke N., Keller S. (2010). Membrane solubilisation and reconstitution by octylglucoside: Comparison of synthetic lipid and natural lipid extract by isothermal titration calorimetry. Biophys. Chem..

[35] Lichtenberg D., Ahyayauch H., Goñi F.M. (2013). The Mechanism of Detergent Solubilization of Lipid Bilayers. Biophys. J..

[36] Homolya L., Holló Z., Germann U.A., Pastan I., Gottesman M.M., Sarkadi B. (1993). Fluorescent cellular indicators are extruded by the multidrug resistance protein. J. Biol. Chem..

[37] Mittra R., Pavy M., Subramanian N., George A.M., O’Mara M.L., Kerr I.D., Callaghan R. (2017). Location of contact residues in pharmacologically distinct drug binding sites on P-glycoprotein. Biochem. Pharmacol..

[38] Rigaud J.-L., Pitard B., Levy D. (1995). Reconstitution of membrane proteins into liposomes: Application to energy-transducing membrane proteins. Biochim. Biophys. Acta.

[39] Ambudkar S.V., Lelong I.H., Zhang J., Cardarelli C.O., Gottesman M.M., Pastan I. (1992). Partial purification and reconstitution of the human multidrug-resistance pump: Characterization of the drug-stimulatable ATP hydrolysis. Proc. Natl. Acad. Sci. USA.

[40] Shapiro A.B., Ling V. (1995). Reconstitution of Drug Transport by Purified P-glycoprotein. J. Biol. Chem..

[41] Dao T.P.T., Brûlet A., Fernandes F., Er-Rafik M., Ferji K., Schweins R., Chapel J.-P., Fedorov A., Schmutz M., Prieto M. (2017). Mixing Block Copolymers with Phospholipids at the Nanoscale: From Hybrid Polymer/Lipid Wormlike Micelles to Vesicles Presenting Lipid Nanodomains. Langmuir.

[42] Magnani C., Montis C., Mangiapia G., Mingotaud A.-F., Mingotaud C., Roux C., Joseph P., Berti D., Lonetti B. (2018). Hybrid vesicles from lipids and block copolymers: Phase behavior from the micro- to the nano-scale. Colloids Surf. B Biointerfaces.

[43] Nam J., Beales P.A., Vanderlick T.K. (2011). Giant Phospholipid/Block Copolymer Hybrid Vesicles: Mixing Behavior and Domain Formation. Langmuir.

[44] Sharom F.J., Yu X., Doige C.A. (1993). Functional reconstitution of drug transport and ATPase activity in proteoliposomes containing partially purified P-glycoprotein. J. Biol. Chem..

[45] Brown M.F. (2012). Curvature Forces in Membrane Lipid–Protein Interactions. Biochemistry.

[46] Lichtenberg D., Ahyayauch H., Alonso A., Goñi F.M. (2013). Detergent solubilization of lipid bilayers: A balance of driving forces. Trends Biochem. Sci..

[47] Niroomand H., Venkatesan G., Sarles S.A., Mukherjee D., Khomami B. (2016). Lipid-Detergent Phase Transitions During Detergent-Mediated Liposome Solubilization. J. Membr. Biol..

[48] Pata V., Ahmed F., Discher D.E., Dan N. (2004). Membrane Solubilization by Detergent: Resistance Conferred by Thickness. Langmuir.

[49] Lambert O., Levy D., Ranck J.-L., Leblanc G., Rigaud J.-L. (1998). A new ‘‘gel-like’’ phase in dodecyl maltoside-lipid mixtures: Implications in solubilization and reconstitution studies. Biophys. J..

[50] Nam J., Vanderlick T.K., Beales P.A. (2012). Formation and dissolution of phospholipid domains with varying textures in hybrid lipo-polymersomes. Soft Matter.

[51] McDevitt C.A., Shintre C.A., Grossmann J.G., Pollock N.L., Prince S., Callaghan R., Ford R.C. (2008). Structural insights into P-glycoprotein (ABCB1) by small angle X-ray scattering and electron crystallography. FEBS Lett..

[52] Pollock N.L., McDevitt C.A., Collins R., Niesten P.H., Prince S., Kerr I.D., Ford R.C., Callaghan R. (2014). Improving the stability and function of purified ABCB1 and ABCA4: The influence of membrane lipids. Biochim. Biophys. Acta.

[53] Poruchynsky M.S., Ling V. (1994). Detection of Oligomeric and Monomeric Forms of P-glycoprotein in Multidrug Resistant Cells. Biochemistry.

[54] Storm J., Modok S., O’Mara M.L., Tieleman D.P., Kerr I.D., Callaghan R. (2008). Cytosolic Region of TM6 in P-Glycoprotein: Topographical Analysis and Functional Perturbation by Site Directed Labeling. Biochemistry.

[55] Raviv Y., Pollard H.B., Bruggemann E.P., Pastan I., Gottesman M.M. (1990). Photosensitized labeling of a functional multidrug transporter in living drug-resistant tumor cells. J. Biol. Chem..

[56] Gulati S., Jamshad M., Knowles T., Morrison K., Downing R., Cant N., Collins R., Koenderink J.B., Ford R.C., Overduin M. (2014). Detergent-free purification of ABC (ATP-binding-cassette) transporters. Biochem. J..

