# Supplementary material for: Characterisation of Hybrid Polymersome Vesicles Containing the Efflux Pumps NaAtm1 or P-Glycoprotein
Source: Polymers (Basel). 2020 May 3;12(5):1049. doi: 10.3390/polym12051049 (PMC7284524; doi:10.3390/polym12051049)
Supplement: Supplementary file 1 [file polymers-12-01049-s001.pdf]

SUPPLEMENTARY DATA

Figure S1. Panels (a-e) show the TX-100 mediated solubilisation of vesicles containing varying proportions of PBd-PEO and POPC. Vesicles with 0 or 25 mol% of polymer displayed two-phase solubilisation profiles. The second phase represented a small component of the absorbance and required concentrations above 1mM TX-100 to achieve complete solubilisation.

In contrast, vesicles containing 50 mol%, or greater, PBd-PEO displayed a simple single phase solubilisation profile. As the proportion of polymer was raised three distinct parameters were altered. First, the amount of detergent required to saturate the vesicle (C_{sat} = upper inflection point) was increased as the proportion of polymer increased; C_{sat} (50% PBd-PEO) = 0.85mM, C_{sat} (75% PBd-PEO) = 1.07mM, C_{sat} (100% PBd-PEO) = 3.89mM. Second, the concentration of TX-100 required to fully solubilise the vesicles (C_{sol} = lower inflection point) was progressively higher as the proportion of PBd-PEO was increased from 50% (C_{sol} = 5.4mM), to 75% (C_{sol} = 7.9mM) to 100% (C_{sol} = 9.7mM). Finally, the profile of the solubilisation phase became gradually greater at the higher polymer composition.

These observations demonstrate that inclusion of polymer provides the vesicles with considerable resistance to surfactant mediated solubilisation.

Panels (f-j) demonstrate the effects of switching the lipid component of vesicles to the more complex crude mixture EcCL. Once again, vesicles with 0-25 mol% PBd-PEO displayed biphasic solubilisation profiles; however, where the proportion of polymer was 50 mol% or greater, a monophasic solubilisation profile was observed. Increasing the proportion of PBd-PEO from 50 (C_{sat} = 0.62mM) to 75 (C_{sat} = 0.85mM) and 100mol % (3.04mM) progressively increased the amount of TX-100 to saturate vesicles. Similarly, higher amounts of TX-100 were required to fully solubilise vesicles with 100% PBd-PEO (C_{sol} = 9.7mM), compared to 75% (C_{sol} = 7.4mM) and 50% (C_{sol} = 5.6mM). Finally, an increase in the slope of the solubilisation profiles was observed as the polymer proportion increased.

Consequently, there are only marginal quantitative differences in the interaction between TX-100 and hybrid vesicles containing POPC or EcCL lipids.

The next series of solubilisation profiles (*Figure S2*) for the two distinct lipid species in vesicles with PBd-PEO was done with the mild non-ionic alkyl-maltoside detergent DDM. *Panels (a-e)* reveal the solubilisation profiles for hybrid PBd-PEO vesicles containing varying amounts of POPC. POPC vesicles with no polymer (*panel a*) displayed an initial

increase in the optical density, which has been suggested to indicate vesicle coalescence. Inclusion of 25mol% PBd-PEO revealed a complex solubilisation that also contained a segment associated with an increase in optical density. Vesicles with 50mol%, or greater, PBd-PEO displayed complex biphasic solubilisation profiles. The vesicles comprising 100mol% polymer revealed a shallow solubilisation profile that occurred over a broad range of DDM concentrations. Consequently, it was particularly difficult to accurately define the point of vesicle saturation with DDM.

Panels (f-j) displayed the solubilisation profiles for vesicles comprising the crude EcCL lipid mixture in conjunction with PBd-PEO. Vesicles composed solely of EcCL did not display the increase in optical density observed with POPC lipids, which has also previously been demonstrated. Vesicles with 25 and 50mol% PBd-PEO (*panels g-h*) were characterised by shallow sigmoidal and monophasic profiles. At higher proportions of PBd-PEO (*panels i-j*), the profiles became biphasic with a decidedly shallow initial phase. Similar to the observations with POPC containing vesicles, precisely assigning a saturating concentration of DDM was difficult.

Overall, the data in *Figures S1-S2* demonstrated that the nature of the lipid species did not greatly affect the solubilisation profiles by detergent. In contrast, the detergent species was associated with a significantly greater effect. Clearly, chemical and physical properties of detergent will impact on its interaction with polymersomes.

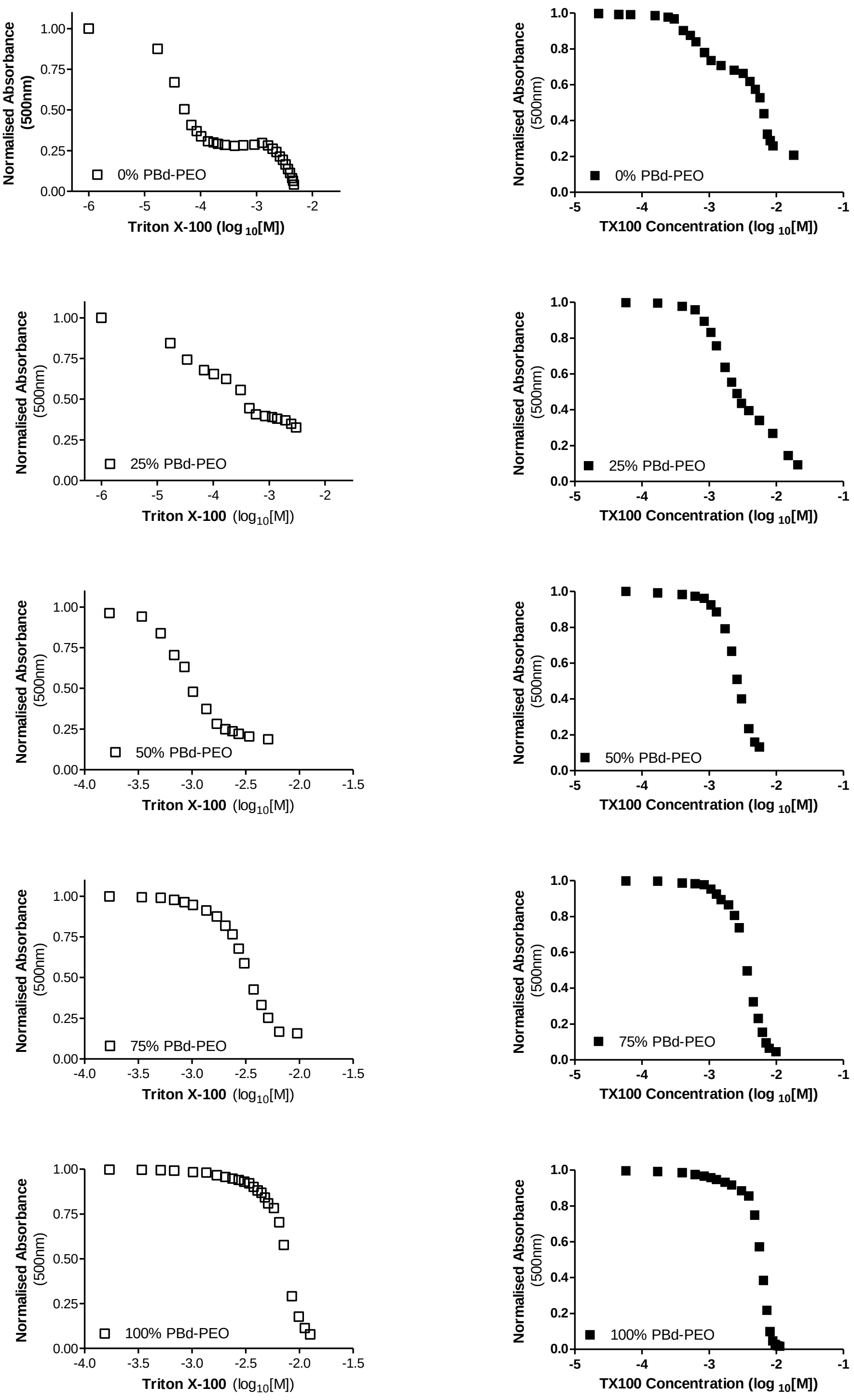

Figure S1 ***Solubilisation of two classes of hybrid polymersome by Triton X-100***

Vesicles containing varying proportions of PBd-PEO and either POPC (a-e) or crude EcCL (f-j) lipids were solubilised by the detergent Triton X-100 (10^{-6} to $10^{-1.5}$M). Percentage proportions of polymer are indicated on each graph. Absorbance (optical density) was measured at 500nm in a 1cm path length quartz cuvette. Detergent was added and signal monitored continuously. Data were normalised to the optical density in the absence of detergent.

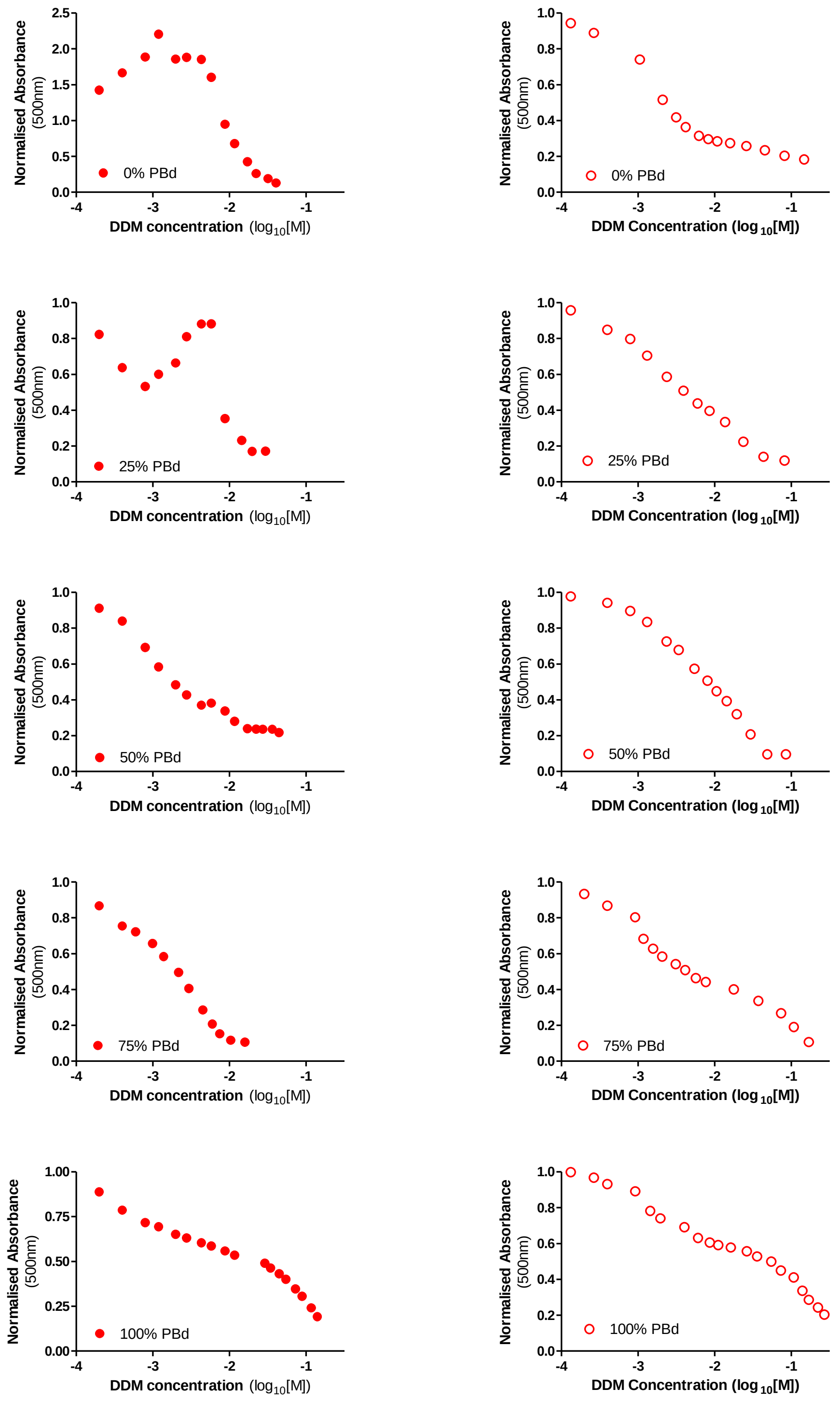

Figure S2 ***Solubilisation of two classes of hybrid polymersome by dodecyl-β-maltoside***

Vesicles containing varying proportions of PBd-PEO and either POPC (a-e) or crude EcCL (f-j) lipids were solubilised by the detergent DDM (10^{-6} to $10^{-1.5}$M). Percentage proportions of polymer are indicated on each graph. Absorbance (optical density) was measured at 500nm in a 1cm path length quartz cuvette. Detergent was added and signal monitored continuously. Data were normalised to the optical density in the absence of detergent.
